# A protocol for analysing the effects on health and greenhouse gas emissions of implemented climate change mitigation actions

**DOI:** 10.12688/wellcomeopenres.16754.1

**Published:** 2021-05-13

**Authors:** Syreen Hassan, Sol Cuevas Garcia-Dorado, Kristine Belesova, Peninah Murage, Sarah Whitmee, Rachel Huxley, Rosemary Green, Andrew Haines

**Affiliations:** 1 Centre on Climate Change and Planetary Health, London School of Hygiene & Tropical Medicine, London, WC1E 7HT, UK; 2 C40 Cities, London, EC4N 4TQ, UK

**Keywords:** Mitigation, implementation, pathways, context, in practice, evaluation, effectiveness

## Abstract

**Background:** It is crucial to understand the benefits to human health from decarbonisation to galvanise action among decision makers. Most of our existing evidence comes from modelling studies and little is known about the extent to which the health co-benefits of climate change mitigation actions are realised upon implementation. We aim to analyse evidence from mitigation actions that have been implemented across a range of sectors and scales, to identify those that can improve and sustain health, while accelerating progress towards a zero-carbon economy.

**Objectives:** To understand the implementation process of actions and the role of key actors; explain the contextual elements influencing these actions; summarise what effects, both positive and negative, planned and unplanned they may have on emissions of greenhouse gases and health; and to summarise environmental, social, or economic co-benefits.

**Data: **We will review evidence collected through partnership with existing data holders and an open call for evidence. We will also conduct a hand search of reference lists from systematic reviews and websites of organisations relevant to climate change mitigation.

**Screening:** Screening will be done by two reviewers according to a pre-defined inclusion and exclusion criteria.

**Analysis**: We will identify gaps where implementation or evaluation of implementation of mitigation actions is lacking. We will synthesise the findings to describe how actions were implemented and how they achieved results in different contexts, identifying potential barriers and facilitators to their design, implementation, and uptake. We will also synthesise their effect on health outcomes and other co-benefits. Quantitative synthesis will depend on the heterogeneity of outcomes and metrics.

**Conclusions: **Findings will be used to identify lessons that can be learned from successful and unsuccessful mitigation actions, to make inferences on replicability, scalability, and transferability and will contribute to the development of frameworks that can be used by policy makers.

## Introduction

It is of great importance to identify and communicate scientific evidence that could support national and sub-national policy makers to take actions towards a post-carbon society, in which there is net zero emissions of carbon dioxide and short-lived climate pollutants. In addition to the benefits of decarbonisation to the environment, there could also potentially be significant benefits to human health ^1, 2^. The Pathfinder Initiative aims to synthesise evidence on the development and implementation of actions across a range of sectors that improve and sustain health while accelerating progress towards a zero-carbon economy. This study will fill a number of knowledge gaps that are impeding progress – namely which climate change mitigation actions will have the largest benefits (and will have the least trade-offs and inequities) for health in particular contexts, what additional environmental, social, or economic benefits such actions might have, and which actions should be employed for effective scale-up in particular contexts. The Pathfinder Initiative seeks to gather and analyse examples where climate change mitigation actions have been implemented in practice and their impacts on health assessed. This protocol describes the process by which these examples will be identified and analysed.

## Aim, objectives, and research questions

The overall aim of the research is to review the evidence from examples where mitigation actions have been implemented *in practice*, in different contexts, and have had an impact on a human health outcomes or exposures. The objectives are: to understand the implementation process of these actions and roles of the key actors; explain the contextual elements influencing these actions; and summarise the impacts these actions might have, both positive and negative, planned and unplanned, on human health. The intention is also to summarise what additional effects they may have on greenhouse gas emissions and other environmental, social, or economic outcomes. We will also seek to identify mechanisms to explain the success or failure of these actions and their implementation, and identify plausible links between actions, context, and outcomes. This study will address the following questions:

### Implementation

- What are the implemented actions and how closely did they correspond to what was intended?
- Who implemented these actions?
- Who are the beneficiaries of these actions (planned and unplanned)?
- Why were these actions implemented?
- Where were those actions implemented?
- How was implementation achieved?
- What are the spatial and timescales of the implemented actions?
- What costs were associated with the implementation?
- What is the potential for scaling up implementation?

### Pathways

- What are the pathways of impact on health?
- What is the response of stakeholders (local, national)?
- Are there unintended consequences (benefits, trade-offs, or spill-over effects)?

### Context

- What contextual elements (barriers and facilitators) influenced the design, implementation, trade-offs and spill-overs, or the rate and scale of uptake of these actions?

### Outcomes and impact

- What is the magnitude of the benefits of these actions on health outcomes?
- What is the magnitude of the benefits, trade-offs, or spill-over effects on other environmental, social, or economic outcomes?
- How are the benefits, and potentially the costs, distributed? Is there equitable distribution across groups, societies and regions?
- What are the timeframes of the benefits achieved or expected to be achieved?

## Design

To answer the research questions outlined above, we will analyse and summarise process and outcome evaluations of mitigation actions that have been implemented in practice. We will also analyse examples where formal evaluations have not taken place or are not explicitly described as such, but where sufficient information is provided to answer the research questions.

We will conduct a thematic synthesis of examples describing the implementation, pathways to impact, and context of implemented mitigation actions. We will describe how these actions achieved their effects in different contexts (including unintended effects and spill overs), or why they did not. This will allow us to develop theories of change that incorporate potential barriers and facilitators of design, implementation, and receipt of these actions, relating to characteristics of participants and contexts.

We will also conduct a narrative synthesis of examples describing outcome evaluations, aimed at summarising measured outcomes and their effects. Where data permits and depending on the heterogeneity of outcome evaluations found, we will conduct a meta-analysis of experimental and quasi-experimental studies of the effectiveness of mitigation actions on health, and any environmental, social, or economic co-benefits to health.

This work will allow us to map which mitigation actions have been implemented in practice and across which sectors and regions and identify where implementation or adequate evaluation of implementation is lacking.

## Data sources

The main source for data will be through a review of evidence, which will be obtained in the following ways.

### First stage of document collection

***Partnership with existing data holders and an open call for evidence.*** We will engage several global collaborators to support data collection: The Organisation for Economic Co-operation and Development (OECD), C40 Cities, the Sustainable Development Solutions Network (SDSN), the Alliance for Health Policy and Systems Research (AHPSR), and the CDP. The OECD has wide expertise in developing and sharing policy analysis and recommendations in relation to climate change, including to negotiators involved in the United Nations Framework Convention on Climate Change (UNFCCC) process. Of particular relevance here, it analyses international policy and practice on climate change mitigation (at national and subnational level) as well as the integration of wider well-being objectives (including health) into climate policy. C40 Cities, through its knowledge hub, has extensive expertise on energy, urban food systems, buildings, transport and urban planning and a large existing data repository of examples of carbon reductions by cities which have benefits for health. The SDSN, which operates under the aegis of the United Nations (UN) Secretary General, has global academic and policy networks across a range of sectors. CDP provides the global platform for over 800 cities and many companies, states and regions to measure, manage and disclose their environmental data every year. The Alliance for Health Policy and Systems Research is an international partnership hosted by the World Health Organisation (WHO) that works to improve the health of those in low- and middle-income countries by supporting the generation and use of evidence that strengthens health systems and is funding collection of examples of mitigation actions with health benefits from several health systems in LMICs.

An open call for evidence will be circulated through networks of the above collaborators and distributed to other international actors including major funders of climate action (e.g., the Green Climate Fund, Regional Development banks, bilateral donors, national and sub-national governments), UN agencies (including WHO and UNDP), the Climate Ambition Alliance, non-governmental organisations (NGOs) and the private sector (through organisations such as the World Business Council on Sustainable Development).

The Pathfinder Initiative also comprises of the Lancet Pathfinder Commission with membership from all major global regions and sectors involved with climate mitigation that provides scientific guidance and oversight. Commissioner networks will be used to further circulate the call. A Comment has been published in The Lancet outlining the Pathfinder Commission and its call for evidence to encourage submissions from readership of The Lancet ^3^.

We will utilise LinkedIn and Twitter in particular for our call for evidence in order to make use of professional networking platforms.

***Drawing on the systematic search for a related study.*** Our second source of evidence will be the collection of systematic reviews identified as part of the umbrella review developed by the Pathfinder Initiative. The umbrella review will meta-synthesise scientific evidence (both modelled and observed in implementation studies) on the solutions/actions that have been synthesised in published systematic reviews (a protocol for this study will be published separately). For the purposes of the analyses described in this protocol, we will review the reference lists and data extracted for the umbrella review to identify any relevant original studies that could serve as further examples meeting our inclusion and exclusion criteria.

***Hand search.*** We will hand search the literature in several ways. First, we will search websites of organisations and climate change projects that are known to the Pathfinder Initiative team, a non-exhaustive list of sources is provided in Table 1. Second, we will examine the reference lists of included documents to identify other potentially relevant documents for inclusion. Finally, we expect that the open call for evidence and our hand search will yield reports that summarise multiple implemented mitigation actions, in addition to reports of implemented mitigation actions that are individually described. Actions discussed in such summary or comprehensive reports will be extracted and traced back to their source for more information.

**Table 1.** List of organisations or projects for website hand search.

African Development Bank
Aga Khan Foundation
Asian Development Bank
Banque de developpement des États de l'Afrique centrale (Development Bank of Central African States)
Banque ouest-africaine de developpement (West African Development Bank)
Bedzed
Central American Bank for Economic Integration
Clean Air Fund
Climate and Clean Air Coalition
Council of Europe Development Bank
Covenant of the Mayors
COWS, Building the High Road
Development Bank of Latin America
Drawdown
Engie
European Commission
European Bank for Reconstruction and Development
Global Alliance on Health and Pollution
Green Climate Fund
GreenWave
Healthcare without Harm
ICLEI – Local Governments for Sustainability
Inter-American Development Bank
International Monetary Fund
Islamic Development Bank Group
NCSE Drawdown conference
Planetary Health Alliance
Solar impulse foundation
SOLVE/MIT
Stockholm Resilience Centre
World Bank
World Resources Institute, Ross Center for Sustainable Cities

### Second stage of document collection

In this stage we will identify gaps in terms of system transitions, as listed in Table 2, as well as world regions, from which we find insufficient evidence and attempt to fill these gaps with a second round of targeted collection. We will ask collaborators, funders of climate change related actions, and members of the Lancet Pathfinder Commission to make suggestions or referrals to relevant local institutions, groups, or communities and take a snowballing approach to identify and purposively target entities within the sectors and regions from which the first stage of document collection yielded insufficient evidence.

**Table 2.** Systems from which to gather evidence on implemented mitigation actions.

Energy System Transitions
Land & Ecosystem Transitions • Food systems (e.g., novel foods and aquaculture)
Industrial System Transitions
Carbon Dioxide Removal
Urban & Infra structure System Transitions • Transport • Healthcare • Education

## Selection criteria

The criteria that we will base our selection of implementation examples on were agreed through discussions among all teams of the Pathfinder Initiative and are as follows.

### Type of document

We will include all types of documents that are submitted to us. This could include published or grey literature, policy documents, internal reports, etc. 

### Year

We will restrict inclusion to examples that describe interventions or actions of climate change mitigation (as defined below), that were implemented from 2000 onwards to keep a relevant scope of actions.

### Language

We will also include reports written in all the languages that are within the capacity of our research team, which includes English, Arabic, French, Spanish, Portuguese, Dutch, Italian, German, Russian, and standard Chinese (Mandarin).

### Implementation stage

We will restrict inclusion to actions that have been fully or partly implemented, including those that are currently ongoing or those that were abandoned due to failures in implementation.

### Actions that achieve climate change mitigation

Based on the Intergovernmental Panel on Climate Change (IPCC) definition, climate change mitigation is defined as actions, or interventions, that reduce the rate of climate change. Climate change mitigation is achieved by limiting or preventing greenhouse gas emissions and short-lived climate pollutants and by enhancing activities that remove these from the atmosphere.

We will include examples where climate change mitigation has been demonstrated through an observed or measured reduction of greenhouse gas emissions and short-lived climate pollutants (as listed in Table 3). We will also include examples of implemented mitigation actions that do not directly measure the reduction in greenhouse gas emissions when there is sufficient evidence for the type of action that mitigation can be assumed. For example, reports that describe a switch from the use of fossil fuels to the use of solar panels as an energy source. Table 4 shows a preliminary list of relevant mitigation actions adapted from the IPCC 2014 Synthesis Report ^4^ that will guide the inclusion and exclusion of implemented mitigation action examples.

We will include all actions where climate change mitigation was achieved, whether as a primary objective, or occurring as one of multiple benefits (whether planned or unplanned) to the implemented action (for example, adaptation or health actions that result in multiple benefits including climate change mitigation).

**Table 3.** list of greenhouse gas emissions and short-lived climate pollutants.

Carbon dioxide (CO _2_)
Carbon monoxide (CO)
Nitrous oxide (N _2_O)
Nitrogen oxides (NOx)
Methane (CH _4_)
Hydrofluorocarbons (HFCs)
Perfluorocarbons (PFCs)
Chlorofluorocarbons (CFCs)
Hydrochlorofluorocarbons (HCFCs)
Sulfur hexafluoride (SF _6_)
Nitrogen trifluoride (NF _3_)
Black carbon
Halocarbons

**Table 4.** Preliminary list of mitigation actions to guide the inclusion / exclusion of examples.

System Category	Action
Energy system transitions	Replace fossil fuel energy with clean energy
	Switch towards renewable energy sources
	Improved energy storage, use and distribution (increased efficiency and flexibility)
	Reduce environmental footprint of fisheries
Land & ecosystem transitions	Shift towards low-carbon diets
	Agro-ecology
	Sustainable agricultural intensification
	Sustainable aquaculture intensification
	Increased energy efficiency of appliances
Industrial system transitions	Switch to materials less intensive in GHG emissions
	Reusing and managing industrial process emissions including methane capture and combustion
	Nature-based solutions
Carbon dioxide removal	GHG capture and storage
	Energy-efficient transportation
Urban & infrastructure system transitions	Reduced demand for travel
	Alternatives to cars
	CNG, Biofuel, Diesel
	Electric/Hydrogen transportation
	Increased energy efficiency of buildings, including insulation and ventilation, to optimise indoor temperature
	Clean cookstoves
	Improved resource management, including recycling
	Reduce consumer waste
	Optimise average house size per person
	Decarbonisation of the healthcare sector
	Economic instruments (taxes, tradable allowances, subsidies)
Cross-cutting strategies	Regulatory approaches
	Information programmes
	Government Provision of Public Goods or Services
	Educational programmes for climate empowerment and behaviour change (reducing food waste and product demand, energy source switch, voluntary family planning especially in contexts with high emissions per capita, optimising indoor temperature)

### Actors

We will include actions implemented by any public sector, civil society, or non-profit actors. Actions that are implemented by individuals or for-profit firms that represent a very large part of a specific sector will be included only if an assessment of impact was conducted by an independent evaluator.

### Implementation scale

We will restrict inclusion to actions that can be adopted by policy-makers and applied on a collective level. This includes plans adopted by national, regional or governmental bodies; international or sectoral agreements; community-led projects; built environments; technologies; governance arrangements; regulatory changes; fiscal mechanisms; mass media interventions; indigenous approaches. We will not include examples of individual or household-level choices which are not described as driven by specific collective or institutional-level actions.

### Health outcomes

**Figure 1.**
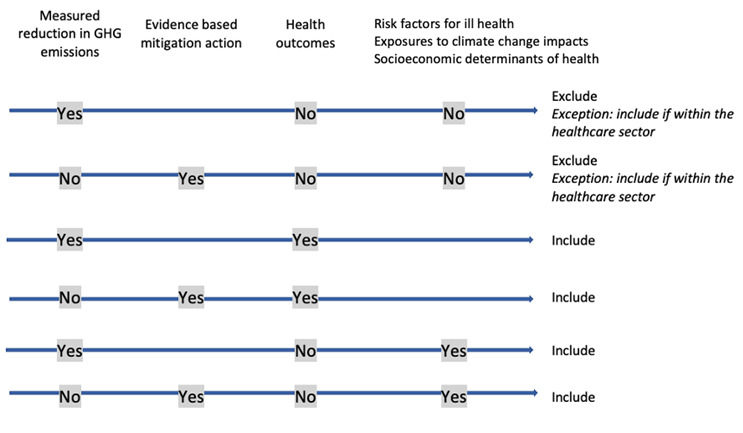
Decision guide for inclusion/exclusion of cases.

We will restrict inclusion to examples of climate change mitigation actions that have demonstrated an observed or measured impact on health outcomes directly, or outcomes that have an evidence-based pathway to health or quantifiable associations to health outcomes. This includes risk factors for health as identified from the Global Burden of Disease ^5^; reduced exposures to climate change impacts (e.g., from combined adaptation and mitigation actions such as reduced heat exposure, or reduced impacts of climate disasters); and improved socioeconomic determinants of health that have direct links to health. A preliminary list of health or health-related outcomes is shown in Table 5. We will also include examples of mitigation actions where no health outcomes are measured only if these are actions implemented within the healthcare sector specifically. In our view, the delivery of healthcare at reduced carbon intensity is a relevant health-related outcome in itself. Figure 1 demonstrates how inclusion or exclusion of examples based on the definition of mitigation actions and health or health-related outcomes will be decided.

**Table 5.** Preliminary list of health outcomes or risk factors for health to guide the inclusion/exclusion of examples.

Health outcomes
Disease manifestations of poor diets (vitamin and mineral deficiencies)
Disease manifestations of air pollution (chronic lung and cardiovascular diseases)
Disease manifestations of increased climate sensitive pathogens (Increase in incidence e.g., malaria, Lyme disease, West Nile virus
Hearing impairment or loss (noise pollution)
Extreme weather-related morbidity/mortality (heat, cold, flooding, droughts, storms, wildfires)
Loss of productivity from weather related morbidity/mortality
Transport related morbidity/mortality (road traffic accidents)
Mental health conditions
Urban heat islands
Risk factors for ill health (GBD)
Childhood underweight
Diet low in fruits, vegetables, legumes, nuts and seeds, seafood and omega-3 fatty acids
Diet high in red meats
Overweight and obesity (BMI)
Physical inactivity
Ambient particulate matter and ozone pollution
Household air pollution exposure
Noise pollution
Chemical pollution
Indoor conditions
Crowding / physical proximity
Exposures to climate change impacts
Disruptions to water supply and quality
Disruptions to energy
Disruptions to healthcare access
Disruptions to food supply
Water, energy, or food security
Displacement
Lower crop yield and loss of livestock
Increase in climate sensitive pathogens and vectors (ticks, mosquitoes, sand flies)
Socio-economic determinants of health
Poverty ^6, 7^
Homelessness ^8, 9^
Unemployment ^10– 12^
Female education and female participation in the workforce ^13^

### Measured impact

We will restrict inclusion to examples that report on measured or observed health outcomes (as defined in the sections above), not those that are solely forecast or modelled.

## Screening

We operationalised the criteria described above into an inclusion and exclusion worksheet ( Table 6) through pilot screening of 20 documents that were already known to the research team. Four reviewers (RG, SH, SCGD, SW) screened the documents in pairs and met to discuss the screening process to ensure consistency in applying the criteria. This resulted in refining the screening criteria and developing the inclusion and exclusion worksheet.

The documents used in the pilot process were not of the typical format of published literature, whereby a clear title and abstract can be used for screening. The reviewers relied on executive summaries for screening, and in the case that executive summaries did not provide enough detail to enable a decision, the full document was assessed. This is expected to be the case for the majority of the full set of documents.

Screening of the full set of documents will be done by two reviewers. One reviewer (SH) will screen all the available documents. Documents will then be divided among the rest of the team for a second screening, blinded to the assessment of the first reviewer. The reviewers will meet to discuss and resolve differences in opinion, and the principal investigators (PI) will make the final decision in case of disagreement.

Screening will be conducted using the screening worksheet on Microsoft Excel (Version 16). Each document will be entered as a row and assessed against each of the criteria presented in the columns ( Table 6). 

**Table 6.** Screening worksheet operationalised from the inclusion exclusion criteria *.

Inclusion or exclusion criteria **									Flags for additional analysis ***				
Language	Implemented after year 2000?	Implementation status	Actors	Mitigation action?	Level of action	GHG emissions	Health	Risk factors to health	Implementation outcomes	Environmental outcomes	Economic outcomes	Social outcomes	Include?
Free text	Yes	Fully implemented	Non-profit actors	Yes	Individual or household- level choices	Measured	Measured (objectively)	Measured (objectively)	Measured (objectively)	Measured (objectively)	Measured (objectively)	Measured (objectively)	Definite Yes
	No	Currently ongoing	For-profit actors with independent evaluation	No	Collective level	-	Measured (self- reported)	Measured (self- reported)	Measured (self- reported)	Measured (self- reported)	Measured (self- reported)	Measured (self- reported)	Definite No
	Not clear	Pilot / Partially implemented	For-profit actors not independently evaluated			Modelled	Modelled	Modelled	Modelled	Modelled	Modelled	Modelled	Possible (need more information)
		Pledged				Measured or modelled but not reported	Measured or modelled but not reported	Measured or modelled but not reported	Measured or modelled but not reported	Measured or modelled but not reported	Measured or modelled but not reported	Measured or modelled but not reported	Not sure
		Planning stage				Not measured or modelled	Not measured or modelled	Not measured or modelled	Not measured or modelled	Not measured or modelled	Not measured or modelled	Not measured or modelled	No but need to check for update on status
						Not clear	Not clear	Not clear	Not clear	Not clear	Not clear	Not clear	
							Modelled on self-reported risk factors						

* This table shows the possible classification, or labelling, that could be given to each example of an implemented mitigation action, for each of the criteria specified in the columns. ** The criteria that will be used to guide the decision on inclusion or exclusion, as described in the ‘selection criteria’ section of this protocol *** Outcomes that will be flagged during the screening process to aid in the identification of these examples for analysis of co-benefits, or additional secondary analysis, but will not be used to guide the decision on inclusion or exclusion

## Data extraction

We will extract data to a worksheet on Microsoft Excel (Version 16) on study characteristics (study location, timing of mitigation action, timing of evaluation); organisational and individual participant characteristics; and characteristics of study design and methods (research aims and objectives, sampling, data collection and analysis). We will use NVivo 12 to code data on implementation, pathways, context, and outcomes.

The Medical Research Council (MRC) guidance for process evaluation of complex interventions ^14^ will be used to guide the extraction of process evaluation data as follows.

**Theory of change:** We will extract data on the theory of change underpinning the implemented action; links to other theories; and the descriptions of how the action is intended to achieve its health, environmental, or socioeconomic outcomes.

**Action design or development:** We will extract data on how the mitigation action was designed or developed and by whom.

**Action characteristics:** We will extract data on the description of the specific climate change mitigation actions that were used; the components; sector or domain; target; and scale of the action.

**Resources:** We will extract data on resources that were required for the implementation of the intervention. These include human, material, and economic resources, including costs of the intervention and costs associated with implementing the intervention including investment, supply, and opportunity costs.

**Implementation:** We will extract data on individual or multifaceted strategies that were used to implement these actions and the broader key processes that were used, such as planning, educating, financing, restructuring, managing quality, or attending to policy context. We will extract data on the fidelity, or the quality, of the implemented action in practice compared to what was designed or intended and whether adaptations had to be made; the extent to which the intended population was reached; the strength of the implemented action, i.e., how much of the intended action was actually delivered; and the acceptability of the action to stakeholders or the target population. We will also extract data on the characteristics of the actors, both on the organisational as well as the individual level, including who enacted the mitigation actions, their role and their skills.

**Context:** We will also extract information on contextual factors affecting the design, communication and implementation of actions, trade-offs and spill overs, and the rate and scale of uptake. This will include contextual factors within the organisation (inner setting), as well as the wider cultural, economic, and governance and political context (outer setting).

For outcome evaluations, we will extract data on the research design; the type of impact; which could be either net impact or gross impact; the outcomes measured (health, environmental, economic); and effect sizes. In the case of experimental or quasi-experimental studies (first tier of evidence), we will extract data on the nature of the control group(s); unit of allocation; generation and concealment of allocation; blinding; adjustment/control of clustering and confounding.

One reviewer (SH) will extract the data. The reviewer will pilot data extraction on 15 studies and discuss their extraction coding with the Pathfinder team to ensure quality and consistency in their interpretation, and will meet regularly with the Pathfinder team to discuss their coding and findings.

We expect that the documents obtained for this study will mainly be in the form of reports, administrative documents or internal records, news articles, etc. and as such are written for a specific purpose and audience other than research. In the case that the documents are found to have missing information that precludes quality assessment or synthesis of findings, we will contact the authors of the reports for additional information.

## Quality assessment

The quality of each included case will be assessed by two reviewers. One reviewer (SH) will appraise all included documents. Documents will then be divided among the rest of the team for a second appraisal. Differences in opinion will be resolved by discussion and in the case of disagreement the PI will make the final decision.

The quality of each study will be assessed based on the AACODS (Authority, Accuracy, Coverage, Objectivity, Date, Significance) checklist that is designed for the critical appraisal of grey literature ^15^. This checklist is used to assess the grey literature based on the following criteria: authority, or who is responsible for the intellectual content; the accuracy of the content, based on whether the document has clear aims and objectives and used valid methodologies that are clearly described; whether limitations in the coverage of the work are clearly stated; the extent to which the work is objective or is biased (i.e., representing opinion); whether the work is clearly dated in terms of when it took place, when it was reported, and key contemporary material were included; and whether the work undertaken is of significance or relevance to the research area.

In addition to this overall assessment, further assessment for each of the process and outcome evaluation data will be undertaken.

### Process evaluations

Documents that report on process evaluations (whether explicitly using this terminology or implicitly describing implementation, pathways of change, and context) will be assessed using quality tools for qualitative studies ^16, 17^. These criteria address reliability in terms of the rigour of sampling, data collection, and data analysis; and usefulness in terms of breadth and depth of findings, and the extent to which stakeholder perspectives were explored ( Table 7).

**Table 7.** Quality assessment of qualitative data.

Rigour
Were there clearly stated aims and objectives?
Were steps taken to minimise bias and error/increase rigour in sampling?
Were steps taken to minimise bias and error/increase rigour in data collection?
Were steps taken to minimise bias and error/increase rigour in data analysis?
Were the findings of the study grounded in/supported by data?
Usefulness
Was there good breadth and/or depth achieved in the findings?
Was there an explicit account of a theoretical framework, a theory of change, or a logic model and/or the inclusion of a literature review which outlined a rationale for the intervention?
Was the implementation of mitigation actions adequately described?
Were the perspectives of stakeholders adequately explored?
Was there a clear description of context which includes detail on barriers and facilitators important for interpreting the results?

### Outcome evaluation

Quantitative studies may be classified according to the following hierarchy of evidence. Studies will be classified into “Tier 1” if they use methods that control for confounders, such as natural experiments, experimental (cluster randomised controlled trials, stepped-wedge trials, etc.) or quasi-experimental designs (interrupted time series, difference in difference, etc.). The strongest type of evidence will be from studies that report on a control or comparison group that is similar in its characteristics and pre-intervention outcome variables to the intervention group and report on pre- and post-intervention data for all groups recruited into the evaluation and on all outcomes. Studies will be classified into a “Tier 2” of evidence when they use a simple comparator, such as before/after measures, or cross-sectional data to compare affected or unaffected areas. Studies will be classified into “Tier 3” of evidence if they report on data collected after the intervention with no comparator provided. Finally, a “Tier 4” of studies will be those that report a description of the health outcomes achieved rather than providing quantitative estimates. The quality of the documents within each of these tiers of evidence will be appraised using standard Critical Appraisal Skills Program tools.

## Synthesis and reporting

We will identify gaps in evidence where we did not find examples of implementation or implementation that have been adequately evaluated against a framework of mitigation actions, health outcomes, and pathways to health outcomes, which is currently under development. The findings will in turn also be used to refine this framework in an iterative process.

We will produce a taxonomy of specific mitigation solutions that have been implemented in practice, group them into broader categories of mitigation actions, and describe how they were implemented, the effects they had on different outcomes, and how they achieved their effects in different contexts. We will identify the potential barriers and facilitators to the design, implementation, and receipt of these actions among the target population. We will compare the types of actions that have been implemented and the context within which implementation took place between examples to identify similarities and differences.

We will conduct a narrative synthesis of the effects of mitigation actions on health outcomes, or any environmental and socioeconomic co-benefits. Once we know the number of implementation examples available to us and the extent of heterogeneity amongst them, we will make a decision on whether and how we can present a pooled effect size. For example, if we find that a sufficient number of examples of similar mitigation actions have measured similar outcomes, we may be able to pool these effects.

We will use graphical synthesis techniques to represent our data. For example, heat maps to show the distribution of evidence across sectors, and harvest plots where quantitative evidence on outcomes is available but not amenable to pooled analysis. 

We will use the findings to identify lessons that can be learned from both successful and unsuccessful implementation of mitigation actions and implementation strategies used to deliver these actions, and make inferences on their replicability, scalability, and transferability. The findings from this synthesis will contribute to the development of frameworks and documents that can be used by policy makers and other actors in the field of planetary health. We will produce practical guidelines targeted towards key audiences with a focus on the findings of this study, illustrating worked examples of what actions can be implemented in practice, how they can be implemented, in which contexts, and what effects they might have on health and other co-benefits.

## Dissemination

Early findings will be published in an interim Commission report in The Lancet ahead of COP26 in November 2021 and a more detailed overview of the key findings will be published in the full Commission report ahead of COP27 in 2022. Example case studies and policy briefs for specific audiences will also be published online and shared through our partner networks in briefing papers, newsletters and through webinars and presentations to key stakeholder groups. Results will also be shared at key events on decarbonisation and health in 2021 and 2022 including (but not limited to) the Planetary Health Alliance annual meetings, the 2021 WHO Global Conference on Climate Change and Health and the World Health Summit in October 2021.

## Study status

We are currently undertaking the first stage of document collection. We are engaging with partners to search their existing databases and have circulated our open call for evidence through our networks of collaborators and other international actors and major funders, and social media, as outlined in the ‘Data sources’ section. We have also identified several documents from the references of the systematic search for a related study and the hand search, as outlined in the ‘Data sources’ section. One reviewer (SH) has screened 30 documents that have been identified from these processes so far.

## Data availability

No data are associated with this article.
