# Supplementary material for: A protocol for analysing the effects on health and greenhouse gas emissions of implemented climate change mitigation actions
Source: Wellcome Open Res. 2022 May 25;6:111. Originally published 2021 May 13. [Version 2] doi: 10.12688/wellcomeopenres.16754.2 (PMC9608249; doi:10.12688/wellcomeopenres.16754.2)
Supplement: Peer review history

# Peer review: Reviewer response for version 2

**DOI:** 10.21956/wellcomeopenres.19814.r50747

**Published:** 2022-06-24

**Authors:** Jose M. Rodriguez-Llanes

**Affiliations:** 1 European Commission Joint Research Centre, Ispra, Italy

I read the revisions and they look fine.

Is the study design appropriate for the research question?

Yes

Is the rationale for, and objectives of, the study clearly described?

Yes

Are sufficient details of the methods provided to allow replication by others?

Yes

Are the datasets clearly presented in a useable and accessible format?

Not applicable

Reviewer Expertise:

NA

I confirm that I have read this submission and believe that I have an appropriate level of expertise to confirm that it is of an acceptable scientific standard.

# Peer review: Reviewer response for version 2

**DOI:** 10.21956/wellcomeopenres.19814.r50746

**Published:** 2022-06-10

**Authors:** Byomkesh Talukder

**Affiliations:** 1 Dahdaleh Institute for Global Health Research (DIGHR), York University, Toronto, ON, Canada; 2 Planetary Health Fellow, Toronto, ON, Canada

I have no further comments to make.

Is the study design appropriate for the research question?

Yes

Is the rationale for, and objectives of, the study clearly described?

Yes

Are sufficient details of the methods provided to allow replication by others?

Yes

Are the datasets clearly presented in a useable and accessible format?

No

Reviewer Expertise:

Planetary Health, Health Impacts of Climate Change, Sustainability Assessment of Food and Agricultural Systems, Systems Thinking, System Dynamics, Indicator Development, Multi-Criteria Decesion Analaysis

I confirm that I have read this submission and believe that I have an appropriate level of expertise to confirm that it is of an acceptable scientific standard.

# Peer review: Reviewer response for version 1

**DOI:** 10.21956/wellcomeopenres.18476.r45445

**Published:** 2021-09-27

**Authors:** Jose M. Rodriguez-Llanes

**Affiliations:** 1 European Commission Joint Research Centre, Ispra, Italy

This study protocol is extremely welcome, and will be helpful in keeping track and accountable the efforts to decarbonize our planet and give birth to a new world economy. This protocol opens an important path, one of demonstrating the multi-layered benefits of mitigation policies.

 The authors are realistic about their prospects for project implementation: selected examples where sufficient evidence is available and have been published will be analyzed via secondary analysis (e.g., meta-analyses) and supplemented with thematic analysis. This is fine. And yet one might question if given the complexity and emergency of the task, should the authors consider running their own integrated statistical analyses using some of these data systems? For example, getting all the evidence together could take considerable time if we think that reports sometimes take years to appear and might be of varying quality. But also understandable, many of these fields are out of reach and use highly specialized data not readily available or even shared. One benefit of integrated analysis is robustness of the findings (given data is jointly analyzed), other is quality control of analytics. By adding this option into their protocol, the authors could answer specific questions, which maybe remain unanswered by others.

**Ending paragraph at Design section, the authors wrote:**

 ‘This work will allow us to map which mitigation actions have been implemented in practice and across which sectors and regions and identify where implementation or adequate evaluation of implementation is lacking’.

 I think this point is contradictory. The intent, if I understand correctly, is to capture examples which can be investigated in-depth, and for which sufficient quality evidence can provide an overall picture of benefits and co-benefits. The sentence above (‘map’) gives the impression that this will be an exhaustive effort.

**Table 5:**

 Shall table 5 include wasting, stunting, and other standard, widely used, and measurable nutritional outcomes? In addition to deficiencies in minerals and vitamins?

**In section Measured impact, the authors wrote:**

 ‘We will restrict inclusion to examples that report on measured or observed health outcomes (as defined in the sections above), not those that are solely forecast or modelled.’

 I assumed that a great majority of those will be modelled observed data, as health outcomes could be confounded by other interventions in the real world (reason why authors rightly chose to include experimental or quasi-experimental studies). Hence, I am confused by the sentence (‘not those that are solely forecast or modelled'). Moreover, in figure 1, the two first situations may include reports with no health outcomes. This seems, at first, to contradict the above statement by the authors.

 Overall, a good piece of work, and a clear guide for the ongoing and future data collection and synthesis. The piece has the potential to inspire more scientific evidence and accelerate the scaling of similar initiatives by other groups.

Is the study design appropriate for the research question?

Yes

Is the rationale for, and objectives of, the study clearly described?

Yes

Are sufficient details of the methods provided to allow replication by others?

Yes

Are the datasets clearly presented in a useable and accessible format?

Not applicable

Reviewer Expertise:

Climate and disaster epidemiology, resilience

I confirm that I have read this submission and believe that I have an appropriate level of expertise to confirm that it is of an acceptable scientific standard, however I have significant reservations, as outlined above.

# Response

**Published:** 2022-05-04

**Authors:** Syreen Hassan

**Affiliations:** London School of Hygiene & Tropical Medicine, UK

Thank you very much for reviewing our article and for your helpful comments.

 In response to your comments: 

 "And yet one might question if given the complexity and emergency of the task, should the authors consider running their own integrated statistical analyses using some of these data systems? For example, getting all the evidence together could take considerable time if we think that reports sometimes take years to appear and might be of varying quality. But also understandable, many of these fields are out of reach and use highly specialized data not readily available or even shared. One benefit of integrated analysis is robustness of the findings (given data is jointly analyzed), other is quality control of analytics. By adding this option into their protocol, the authors could answer specific questions, which maybe remain unanswered by others."

 Thank you for your comments you’re your suggestion. We do aim to synthesise and integrate the findings from all literature that we find (whether reports or published articles), in an iterative manner. That is, analysis will begin alongside data collection, whereby early findings will feed into how we collect data and which areas we target, and the additional data collected will be added and analysed. Therefore, findings will be synthesised and integrated as we go along, rather than wait until data collection is complete.

 "Ending paragraph at Design section, the authors wrote: ‘This work will allow us to map which mitigation actions have been implemented in practice and across which sectors and regions and identify where implementation or adequate evaluation of implementation is lacking’. I think this point is contradictory. The intent, if I understand correctly, is to capture examples which can be investigated in-depth, and for which sufficient quality evidence can provide an overall picture of benefits and co-benefits. The sentence above (‘map’) gives the impression that this will be an exhaustive effort."

 Thank you for raising this point. We have made a change taking this into account.

**"Table 5:**

 Shall table 5 include wasting, stunting, and other standard, widely used, and measurable nutritional outcomes? In addition to deficiencies in minerals and vitamins?"

 The table is not intended to be exhaustive. As the title of the table “Preliminary list of health outcomes or risk factors for health to guide the inclusion/exclusion of examples” suggests **, t**his list is preliminary because it will be updated with any additional outcomes that are identified from the literature, and it was put together only to guide data extraction and not to be prescriptive. We do mention that data extraction and analysis will be iterative.

**"In section Measured impact, the authors wrote:**

 ‘We will restrict inclusion to examples that report on measured or observed health outcomes (as defined in the sections above), not those that are solely forecast or based on data that are assumed.’ I assumed that a great majority of those will be modelled observed data, as health outcomes could be confounded by other interventions in the real world (reason why authors rightly chose to include experimental or quasi-experimental studies). Hence, I am confused by the sentence (‘not those that are solely forecast or modelled')."

 Thank you, we have made a clarification. We do not mean that we will not use analytical models. We only mean that outcomes should be observed, rather than forecast or based on assumed data.

 "Moreover, in figure 1, the two first situations may include reports with no health outcomes. This seems, at first, to contradict the above statement by the authors."

 We do make clear in the text that we make an exception for actions that aim to decarbonise the healthcare sector, and this is the only exception we make.

# Peer review: Reviewer response for version 1

**DOI:** 10.21956/wellcomeopenres.18476.r45069

**Published:** 2021-09-16

**Authors:** Byomkesh Talukder

**Affiliations:** 1 Dahdaleh Institute for Global Health Research (DIGHR), York University, Toronto, ON, Canada; 2 Planetary Health Fellow, Toronto, ON, Canada

- From the systems thinking perspective, the authors can rewrite the introduction to establish the linear and nonlinear interconnectedness of the effects on health and greenhouse gas emissions of implemented climate change mitigation actions. This will give a solid background of the purpose of the protocol.
- This paper requires a fugue to illustrate the research questions and the methods to answers those questions and probable outcomes.
- “Table 4. Preliminary list of mitigation actions to guide the inclusion/exclusion of examples” needs to be reorganized - some actions do not fit with the systems category such as in “Energy system transitions” the authors listed “Reduce the environmental footprint of fisheries” as action. I do not understand it. Please check this table very carefully.
- The authors can add the probable outcomes of this protocol. This can be discussed in the discussion section.
- If it is a manuscript, it also requires a conclusion.
- The authors have presented a lot of information in the manuscript. The authors can be more sequential and logical to present their information.

Is the study design appropriate for the research question?

Yes

Is the rationale for, and objectives of, the study clearly described?

Yes

Are sufficient details of the methods provided to allow replication by others?

Yes

Are the datasets clearly presented in a useable and accessible format?

No

Reviewer Expertise:

Planetary Health, Health Impacts of Climate Change, Sustainability Assessment of Food and Agricultural Systems, Systems Thinking, System Dynamics, Indicator Development, Multi-Criteria Decesion Analaysis

I confirm that I have read this submission and believe that I have an appropriate level of expertise to confirm that it is of an acceptable scientific standard, however I have significant reservations, as outlined above.

# Response

**Published:** 2022-05-04

**Authors:** Syreen Hassan

**Affiliations:** London School of Hygiene & Tropical Medicine, UK

From the systems thinking perspective, the authors can rewrite the introduction to establish the linear and nonlinear interconnectedness of the effects on health and greenhouse gas emissions of implemented climate change mitigation actions. This will give a solid background of the purpose of the protocol.

 Thank you for your comment. We recognise that there is an interconnectedness of the effects on health and greenhouse gas emissions of implemented climate change mitigation actions. However, for this piece of work we are aiming identify actions that are achieve reductions in greenhouse gas emissions, as well as benefits for health. The focus here is on co-benefits, and the pathways of change that demonstrate how a mitigation action can also achieve benefits for health, in addition to mitigation of climate change, not the benefits to health that result from mitigation of climate change. We have made slight change to the text in response to this.

 This paper requires a figure to illustrate the research questions and the methods to answers those questions and probable outcomes.

 Thank you for this comment. We have considered it carefully and decided that a figure will only be repetitive of what is in the text and the tables that are already provided. We list all the research questions under the heading “Aim, objectives, and research questions”. To answer the research questions relating to impact, we provide Table 4 to list the greenhouse gases that we seek to find reported reductions on and Table 5 to list the potential health outcomes that may be reported. We also list all the themes that we wish to extract data on (under data extraction) to answer research questions relating to implementation, pathways, context.  

 “Table 4. Preliminary list of mitigation actions to guide the inclusion/exclusion of examples” needs to be reorganized - some actions do not fit with the systems category such as in “Energy system transitions” the authors listed “Reduce the environmental footprint of fisheries” as action. I do not understand it. Please check this table very carefully.

 This is an error in print, rather than how the table is supposed to be organised. We have fixed this error.

 The authors can add the probable outcomes of this protocol. This can be discussed in the discussion section.

 We have listed probable health outcomes in Table 5. We also list the probable themes we aim to extract data on in the section “Data extraction”.
